# Emergence of Epidemic Zika Virus Transmission and Congenital Zika Syndrome: Are Recently Evolved Traits to Blame?

**DOI:** 10.1128/mBio.02063-16

**Published:** 2017-01-10

**Authors:** Scott C. Weaver

**Affiliations:** Institute for Human Infections and Immunity and Department of Microbiology and Immunology, University of Texas Medical Branch, Galveston, Texas, USA

## Abstract

The mechanisms responsible for the dramatic emergence of Zika virus (ZIKV), accompanied by congenital Zika syndrome and Guillain-Barré syndrome (GBS), remain unclear. However, two hypotheses are prominent: (i) evolution for enhanced urban transmission via adaptation to mosquito vectors, or for enhanced human infection to increase amplification, or (ii) the stochastic introduction of ZIKV into large, naive human populations in regions with abundant *Aedes aegypti* populations, leading to enough rare, severe infection outcomes for their first recognition. Advances in animal models for human infection combined with improvements in serodiagnostics, better surveillance, and reverse genetic approaches should provide more conclusive evidence of whether mosquito transmission or human pathogenesis changed coincidentally with emergence in the South Pacific and the Americas. Ultimately, understanding the mechanisms of epidemic ZIKV emergence, and its associated syndromes, is critical to predict future risks as well as to target surveillance and control measures in key locations.

## COMMENTARY

Zika virus (ZIKV) has emerged over the past 10 years to cause outbreaks of human infection in the Pacific Islands followed by the Americas, where it is poised to cause millions of infections. Most of these infections are asymptomatic, and the majority of symptomatic cases are mild with rash, conjunctivitis, joint pains, and fever. However, infection can occasionally lead to Guillain-Barré syndrome (GBS), a paralytic disease of the peripheral nervous system, or a wide range of congenital defects and malformations, including microcephaly, when a pregnant woman is infected (1). Because these severe outcomes of ZIKV infection as well as the magnitude of the outbreaks in French Polynesia and the Americas are unprecedented, explanations for the recent emergence have mainly focused on two hypotheses.

### (i) Adaptive evolution.

In a recent article, Pettersson et al. (2) addressed the hypothesis that the recent emergence of ZIKV epidemic transmission and congenital Zika syndrome is associated with genetic changes in the virus that accompanied its spread to the South Pacific and subsequently to Latin America.

**FIG 1:**
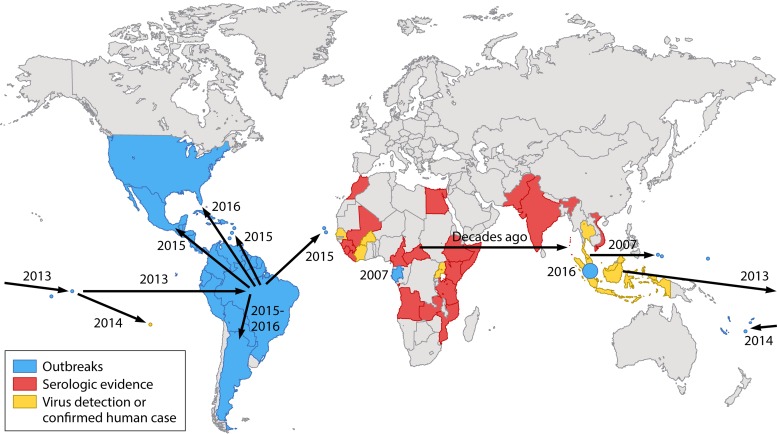
Map of ZIKV evolution in sub-Saharan Africa, spread to Asia, and introductions into Yap Island, the South Pacific, and the Americas inferred from phylogenetic data. Adapted from reference 3 with permission.

Like dengue virus (DENV) and chikungunya virus (CHIKV), ZIKV appears to have originated in zoonotic transmission cycles involving nonhuman primates and arboreal mosquito vectors (3); also like CHIKV, ZIKV evolved and was first discovered in sub-Saharan Africa (Fig. 1). The divergence of the African and Asian lineages (4) probably occurred many decades ago, with the spread beyond Southeast Asia to Pacific islands first detected in 2007 in Yap (5). Pettersson et al. (2) hypothesize that, also like CHIKV (6), ZIKV may have recently undergone adaptive evolution for enhanced transmission by urban mosquito vectors, possibly accompanied by increased human pathogenesis. Using phylogenetic analyses, amino acid changes associated with the emergence of ZIKV from Africa into Asia since the mid-20th century were assigned to tree branches representing different phases of virus movement. These analyses logically emphasize those amino acid changes accompanying the spread into French Polynesia, where the first association between ZIKV infection and Guillain-Barré syndrome (GBS) (7), as well as congenital microcephaly (8), occurred.

Adaptive evolution for enhanced infection of vectors or vertebrate hosts by arboviruses is expected to be mediated mainly by amino acid substitutions in viral proteins. Several substitutions in the ZIKV premembrane protein previously discussed as potentially altering its structure (9) were found to occur on tree branches not leading to the French Polynesian strains, disputing their putative role in epidemic emergence or the evolution of severe fetal infections. Instead, using highly detailed phylogenetic reconstructions, Pettersson et al. discuss a series of mutations believed to have occurred in Southeast Asia in a region of the ZIKV matrix (M) and envelope (E) glycoprotein gene (domain III, believed to be responsible for interactions with cellular receptors of both vertebrate hosts and mosquito vectors). One or more of these E mutations could be responsible either for enhanced transmission by the primary epidemic mosquito vector, *Aedes aegypti*, or for altered human tropism that could explain fetal infection and congenital pathogenesis. One of these mutations, encoding an S139N substitution in the transmembrane M protein, has been identified as an important determinant of viral maturation for the closely related flavivirus DENV (10). Thus, all ZIKV strains associated with epidemic urban, mosquito-borne transmission; sexual transmission via semen; GBS; and congenital Zika syndrome include this M protein substitution along with D683E in the envelope glycoprotein and V763M and T777M in the transmembrane domain of E. Another substitution, M/T2634V, likely occurred after ZIKV spread from French Polynesia or another island in the South Pacific to the Americas (estimated from August 2013 to April 2014) and thus could be associated with the higher incidence of microcephaly in Brazil than in the South Pacific outbreaks (8, 11). Pettersson et al. also suggest that other contemporaneous substitutions in the NS4B and NS5 proteins involved in perturbation of the vertebrate interferon response could also be responsible for recent changes in human pathogenesis. Another recent finding, that ZIKV is significantly more thermostable than DENV, may also be involved in its epidemic spread by virtue of its ability to replicate to higher levels of viremia accompanied by fever and to resist inactivation under unfavorable conditions (12).

### (ii) Stochastic introduction.

In addition to the adaptive evolution hypothesis described above, a competing hypothesis is that ZIKV was simply transported stochastically to locations with large-enough naive human populations, accompanied by large urban mosquito vector populations, for an explosive outbreak. Under these epidemic conditions, formerly rare conditions were suddenly recognized when large enough numbers of infections occurred.

### Prospects and means to test these hypotheses.

Thorough testing of these two competing hypotheses for ZIKV emergence should be a high priority. Potentially altered roles of most viral proteins based on amino acid changes, such as those identified by Pettersson et al. (2) associated with ZIKV spread from Southeast Asia to the South Pacific and then on to the Americas, cannot be predicted with certainty. However, reverse genetic approaches are now available to assess the phenotype of each. Fortunately, cDNA-based clones for ZIKV are available to initiate these studies (13).

Mutations that may affect infection and transmission by the urban vector, *A. aegypti*, can easily be tested using experimental infections with artificial blood meals or viremic mice and wild-type, field-collected or low-generation-colonized mosquitoes. Even simpler experiments comparing African, Asian, and American ZIKV strains have thus far shown the African strains to be fitter for infection of *A. aegypti* mosquitoes (14) associated with transmission in the Americas (15), a finding incompatible with major adaptation for transmission by this vector.

However, assessing potential effects on human pathogenesis will be much more challenging due to the limited animal models available. Wild-type mice can be infected with ZIKV but develop no signs of disease and little or no viremia, limiting their value. Genetically modified mice defective for type I and/or type II interferon signaling, or wild-type mice with the interferon response suppressed by antibodies, develop more systemic infections with viremia; neurologic disease; high viral loads in the brain, spinal cord, and testes; and age-dependent mortality (16, 17). Infection of these mice early during pregnancy results in infection of the placenta and fetal brain, causing fetal disease and spontaneous abortion sometimes observed in ZIKV-infected pregnant women (18). However, these murine models are far from ideal based on their lack of an intact innate immune response and the much higher rates of central nervous system (CNS) and fetal disease than those associated with human infection, especially in the adult stages. Nonhuman primates, often the best models for human viral infections, appear to be more promising. In addition to generating viremia and viral loads in saliva, urine, and cerebrospinal fluid of rhesus macaques in the absence of overt disease (19), ZIKV infection of a pregnant pigtail macaque has produced fetal brain lesions (20).

Testing the stochastic introduction hypothesis will need to rely both on the findings of the experimental work to examine adaptive evolution and on surveillance and epidemiological studies. If Southeast Asian and potentially even African ZIKV strains have the same potential emergence and pathogenesis phenotypes, these findings would support the stochastic hypothesis: that ZIKV amplification in French Polynesia, followed by further spread via air travel into larger naive populations in the Americas, resulted in massive epidemics with the opportunity to detect rare, severe outcomes of infection. Recent reports of *A. aegypti*-borne epidemic ZIKV transmission in Singapore (21) and two cases of ZIKV-associated microcephaly in Thailand (22) suggest that no major phenotypic change in these properties occurred since the ZIKV spread from Asia. Prior to 2003 and even before 2013, there was a lack of attention and efficient diagnostics for ZIKV infection in Asia. Combined with the typical default clinical diagnosis in the absence of laboratory diagnostics of dengue for any arboviral infection producing a nonspecific acute febrile illness in that region, this calls into question the assumption that efficient urban transmission and CNS disease were absent from Asia in the past. Now that commercial diagnostics for ZIKV and antibody responses are becoming available, increased attention and diagnostic attempts may be detecting small outbreaks and congenital infections that have occurred all along. Also, the presence of nearly continuous, low-level endemic transmission in Asia (like that of CHIKV between the 1960s and 1996) may result in levels of herd immunity that limit the efficiency of mosquito-borne transmission and the number of maternal infections to a degree where the incidence of microcephaly above baseline levels caused by other infections can be overlooked, as initially occurred in French Polynesia despite more than 100,000 cases in 2013 to 2014 (8). Even more limited diagnostic capabilities in many parts of Africa could easily mask these same conditions. With the increased availability of ZIKV diagnostics (albeit with remaining limitations on serodiagnosis in locations where dengue is endemic), improved surveillance and epidemiological studies in Southeast Asia and Africa should be able to provide further data to more robustly test the second hypothesis that recent emergences were stochastic, relying on increased air travel and the arrival of ZIKV in regions with completely naive human populations.

Determining the mechanisms of epidemic ZIKV emergence, especially of the appearance of congenital Zika syndrome and GBS, is critical to predict future risks as well as to target surveillance and control measures in key locations. For example, if epidemic potential and adverse infection outcomes have recently evolved with spread to the South Pacific and the Americas, Asia and Africa may be at risk mainly from strains imported from these epidemic locations. Thanks to the ability of phylogenetic methods to reconstruct patterns of ZIKV evolution and individual mutations associated with spread and emergence (2), combined with reverse genetic systems (13), the adaptive evolution hypothesis for ZIKV emergence can now be tested experimentally in relatively short order. Improved animal models for congenital Zika syndrome and eventually for GBS can enhance this opportunity. Also, thanks to international attention on ZIKV leading to increased surveillance in Asia and hopefully also in Africa, the urban transmission and pathogenesis phenotypes of the African and Asian ZIKV lineages should be revealed in the coming years.

A major limitation on the latter analyses is the lack of specificity of serologic tests for past infection with ZIKV. Cross-reactions with antibodies induced by DENV, yellow fever virus, and other flaviviruses continue to limit the ability to assess herd immunity and to accurately diagnose many ZIKV infections. Improvements in serodiagnostics remain a critical need for understanding ZIKV emergence and also determining likely patterns of future spread and infection that can inform optimal control strategies.
